# Factors Associated With Prolonged Psychological Distress Among Nurses and Physicians Engaged in COVID-19 Patient Care in Singapore and Japan

**DOI:** 10.3389/fpsyt.2022.781796

**Published:** 2022-04-28

**Authors:** Shinichiro Morioka, Ban Hock Tan, Hiroe Kikuchi, Yusuke Asai, Tetsuya Suzuki, Shinobu Ashida, Satoshi Kutsuna, Sho Saito, Kayoko Hayakawa, Thuan Tong Tan, Eiichi Kodama, Norio Ohmagari

**Affiliations:** ^1^Disease Control and Prevention Center, National Center for Global Health and Medicine Hospital, Tokyo, Japan; ^2^Emerging and Reemerging Infectious Diseases, Graduate School of Medicine, Tohoku University, Sendai, Japan; ^3^AMR Clinical Reference Center, National Center for Global Health and Medicine Hospital, Tokyo, Japan; ^4^Department of Infectious Diseases, Singapore General Hospital, Singapore, Singapore; ^5^Department of Psychosomatic Medicine, National Center for Global Health and Medicine Hospital, Tokyo, Japan; ^6^Department of Infection Control, Graduate School of Medicine, Osaka University, Suita, Japan; ^7^Division of Infectious Diseases, International Institute for Disaster Science, Graduate School of Medicine, Tohoku Medical Megabank Organization, Tohoku University, Sendai, Japan

**Keywords:** cross-sectional survey, coronavirus disease 2019, pandemic, healthcare providers, mental health, quarantine, psychological distress

## Abstract

This study explores the factors contributing to the prolonged psychological distress of frontline nurses and physicians caring for COVID-19 patients in hospitals in Singapore and Japan. A cross-sectional survey between September and December 2020 yielded 1,644 responses (23.8%), from 62 nurses and 64 physicians in Singapore and 1,280 nurses and 238 physicians in Japan. Multivariate logistic regression analysis revealed that significant risk factors for prolonged psychological distress included being a frontline nurse [adjusted odds ratio (aOR) = 2.40, 95% confidence interval (CI): 1.24–4.66], having an underlying medical condition (aOR = 1.74, 95% CI: 1.22–2.46), experiencing prejudice because they undertook COVID-19 patient care (aOR = 3.05, 95% CI: 2.23–4.18), having trouble dealing with panicked or uncooperative patients (aOR = 2.36, 95% CI: 1.71–3.25), and experiencing an outbreak of COVID-19 in the hospital (aOR = 2.05, 95% CI: 1.38–3.04). Factors inversely associated with psychological distress included age (OR = 0.98, 95% CI: 0.97–1.00), number of beds in the hospital (aOR = 0.73, 95% CI: 0.57–0.94), clinical practice of carefully putting on and taking off personal protective equipment in daily COVID-19 patient care (aOR = 0.52, 95% CI: 0.37–0.73), and knowledge on COVID-19 (aOR = 0.82, 95% CI: 0.72–0.94). These results could help us identify vulnerable healthcare providers who need urgent mental care during the COVID-19 pandemic. Measures that may reduce psychological strain include adequate supply of medical resources, education on precautionary measures, and communication strategies to combat discrimination against frontline healthcare providers.

## Introduction

During the lengthy coronavirus disease 2019 (COVID-19) pandemic, frontline medical professionals, including the nurses and physicians attending patients with COVID-19, were found to have a high risk of mental health problems such as depression, anxiety, insomnia, sleep problems, and posttraumatic stress reactions (1–8). In addition, burnout and stigmatization were prevalent among healthcare workers (3). Despite the frequency of mental health problems among patients and healthcare workers during large outbreaks, few health professionals receive any training in mental health care and coping with stress (9). Although elevated anxiety symptoms and stress coping responses are expected during extraordinary circumstances, there is a risk of an increased prevalence of clinically relevant numbers of people with anxiety, depression, and those who engage in harmful behaviors such as suicide and self-harm. However, a rise in suicide is notably not inevitable, especially with national mitigation efforts (10).

Psychological interventions that target high-risk populations with severe psychological distress are urgently needed (11). Previous reports have identified the main target population for mental care by exploring the risk factors of acute-phase psychological distress for the healthcare workers caring for patients with COVID-19 (1, 9, 11–18). However, few studies have identified the risk factors for prolonged psychological distress during the lengthy COVID-19 pandemic. This study aimed to explore the factors that have contributed to the prolonged psychological distress of the frontline nurses and physicians caring for COVID-19 patients in hospitals in Singapore and Japan.

## Methods

This study was designed as a nationwide, cross-sectional survey in Singapore and Japan. An online self-report questionnaire was sent to eligible nurses and physicians from September 2020 to December 2020 with two reminders 2 weeks and 1 month later. Potential participants were recruited from among the frontline nurses and physicians who cared for patients with COVID-19 in Singapore and Japan. The frontline nurses and physicians were recruited from 370 hospitals designated for infectious diseases in Japan and one hospital in Singapore. The researchers sent the online self-report questionnaire to representatives in the participating hospitals, and the representatives disseminated the questionnaire to the participants. The questionnaires were sent via emails containing a link to the questionnaire. Participation in this survey was voluntary and anonymous. Participants were requested to complete the questionnaire after providing e-informed consent, and no survey incentives were offered. The Singhealth Centralized Institutional Review Board approved the study (2020/2807) in Singapore, and the Institutional Review Board at the National Center for Global Health and Medicine approved the study (NCGM-G-003562-00) in Japan.

### Questionnaire

The questionnaire was developed through a systematic literature review of similar previous studies (1, 9, 11–18) and comprehensive focus group discussions among the authors. We attempted to minimize the number of questions in order to maximize the response rate and to minimize participants' distress. The instrument was piloted on 11 nurses and physicians in Singapore and 15 nurses and physicians in Japan who were involved in caring for patients with COVID-19, who provided feedback on the content, clarity, and format of the items. Minor revisions were made based on feedback.

#### The Psychological Distress of Frontline Nurses and Physicians

The Kessler Psychological Distress Scale (K10) (19) was used to assess the psychological distress of participants. We used the original version of the K10 in Singapore, and we used a Japanese version, which was translated by a certified translation service (Editage), in Japan. As the primary endpoint, the prolonged psychological distress of frontline nurses and physicians was assessed and defined as a K10 score ≥ 30 when psychological distress was the greatest and during the last 30 days of the survey. The secondary endpoint was the K10 score when the psychological distress peaked.

#### Period When Psychological Distress Was the Greatest

The numbers of newly confirmed cases of the first COVID-19 wave in Singapore and Japan were 1,426 and 701, respectively, and peaked on April 21, 2020 and April 11, 2020, respectively. The time from January 2020 to September 2020 was divided into eight periods (1 month each), A to H, using the first peak of the number of newly confirmed cases as a dividing line. Participants identified a period when the psychological distress was the greatest by choosing a point between A and H.

#### Potential Determinants of Psychological Distress of Frontline Nurses and Physicians

We explored four categories as potential determinants of such psychological distress: nurses' and physicians' demographic characteristics, nurses' and physicians' experiences regarding COVID-19 patient care, nurses' and physicians' clinical practice in daily COVID-19 patient care, and nurses' and physicians' knowledge of COVID-19 (1, 9, 11–18).

##### Demographic Characteristics

The participants' characteristics included age, sex, years of clinical experience, profession, specialty, country (Singapore or Japan), medical history, practice setting, and participation in personal protective equipment (PPE) donning/doffing training before the COVID-19 pandemic.

##### Experiences of COVID-19 Patient Care

The respondents shared their experiences with COVID-19 patients through these four statements: (1) I had trouble dealing with panicked patients or patients who did not cooperate with the quarantine; (2) I felt that people were prejudiced against my family and I because I was involved in COVID-19 patient care; (3) My patients died of COVID-19; and (4) there was an outbreak of COVID-19 in the hospital.

##### Clinical Practice in Daily COVID-19 Patient Care

The following three statements were used to assess clinical practice in daily COVID-19 patient care:

(1) I practiced hand hygiene (i) 0–20%, (ii) 21–40%, (iii)

41–60%, (iv) 61–80%, or (v) 81–100%.

(2) I carefully wore and removed my PPE (i) 0–20%, (ii)

21–40%, (iii) 41–60%, (iv) 61–80%, or (v) 81–100%.

(3) I continued to self-quarantine by being apart from family

(i) 0–20%, (ii) 21–40%, (iii) 41–60%, (iv) 61–80%, or (v) 81–

100%.

##### Knowledge on COVID-19

The respondents were asked to choose true or false for seven statements to assess their knowledge on COVID-19: (1) Health care providers should use an N95 mask or higher-level respirator when performing aerosol generation procedures such as intubation, wiping patients, and changing diapers (false). (2) When aerosol-generating procedures are performed and there is a shortage of N95 masks, N95 masks can be reused by covering them with a face shield or a surgical mask (true). (3) Once COVID-19 is completely ruled out, airborne and contact prevention as well as standard precautions can be terminated (false). (4) People with COVID-19 are not infectious before symptom onset, but they often become infectious on the second to third day after symptom onset (false). (5) A negative severe acute respiratory syndrome coronavirus 2 (SARS-CoV-2) polymerase chain reaction (PCR) test result can rule out COVID-19 (false). (6) SARS-CoV-2 can be detected in the respiratory droplets, stools, and sweat of an infected patient (false). (7) Since alcohol is not effective against the new coronavirus, it is important to wash your hands with running water and soap (false). The average score of the seven questions was regarded as a potential contributing factor to the psychological distress of frontline nurses and physicians.

### Statistical Analysis

The respondents' characteristics, psychological distress, experiences regarding COVID-19 patient care, clinical practice in daily COVID-19 patient care, and knowledge of COVID-19 are expressed as median, interquartile range (IQR), or % (*n*), where applicable. Non-parametric statistics were used when variables did not seem to have normal distribution. Data were analyzed using SPSS® Statistics version 25.0 software (IBM®, Armonk, NY, USA).

#### Primary Endpoint

The prolonged psychological distress of frontline nurses and physicians was defined as a K10 score ≥ 30 when psychological distress was the greatest and during the last 30 days of the survey (20). We conducted a multivariate logistic regression analysis to calculate the adjusted odds ratio (aOR) with 95% confidence interval (CI) for the prolonged psychological distress of frontline nurses and physicians. We included the respondents' characteristics, experiences regarding COVID-19 patient care, clinical practice in daily COVID-19 patient care, and knowledge of COVID-19 as independent variables.

#### Secondary Endpoint

We conducted a multivariate linear regression analysis to identify the factors associated with peaks in respondent psychological distress among frontline nurses and physicians. The level of significance for all statistical tests was α = 0.05. We included the respondents' characteristics, experiences regarding COVID-19 patient care, clinical practice in daily COVID-19 patient care, and knowledge of COVID-19 as independent variables. Then, we exploratorily described the relationship between the number of new confirmed cases and the number of participants whose psychological distress was the greatest in each period (A-H).

## Results

A total of 6,915 nurses and physicians were invited to participate in the survey. These included 432 nurses and 527 physicians at the Singapore General Hospital and 4,530 nurses and 1,426 physicians in 370 hospitals in Japan. Of those, 1,644 completed the survey, with an overall response rate of 23.8%. The number of respondents were as follows: 62 nurses and 64 physicians at the Singapore General Hospital and 1,280 nurses and 238 physicians in Japan. The response rates in Singapore and Japan were 13.1 and 25.5%, respectively. The respondents' background characteristics are summarized in Table 1. The missing values ranged between 0 and 0.24%. The median age of the 1,644 respondents was 37 years; 23.4% were male, and the median number of years of clinical experience was 13. Physicians comprised 302 respondents (18.4%) and 1,342 (71.6%) were nurses.

**Table 1:** Characteristics of respondents (*n* = 1,644).

	**Singapore General**	**Japan**
	**Hospital (*n* = 126)**	**(*n* = 1,518)**
	***n* (%)**	***n* (%)**
Age (median, IQR)	34, 29–42	38, 29–46
**Sex**		
Male	32 (25.4)	352 (23.2)
Female	93 (73.8)	1,152 (76.0)
Prefer not to say	1 (0.8)	13 (0.9)
**Profession**		
Nurse	62 (49.2)	1,280 (84.3)
Physician	64 (50.8)	238 (15.7)
Years of clinical experience (years, median, IQR)	10, 5–16	13, 6–23
Living with family	103 (81.7)	1,066 (70.2)
Living with a child under 16 years old	33 (26.2)	486 (32.0)
Living with a person aged 65 or older	42 (33.3)	292 (19.2)
Underlying medical condition	24 (19.0)	379 (25.0)
Hypertension	5 (4.0)	104 (6.9)
Diabetes mellitus	3 (2.4)	16 (1.5)
Bronchial asthma	3 (2.4)	117 (7.7)
Chronic obstructive pulmonary disease	0 (0.0)	0 (0.0)
Heart disease	11 (8.7)	12 (0.8)
Chronic kidney disease	0 (0.0)	3 (0.2)
Malignancy	0 (0.0)	18 (1.2)
Obesity	1 (0.8)	62 (4.1)
Mental illness	0 (0.0)	17 (1.1)
Others	13 (10.3)	122 (8.0)
**Workplace**
Emergency room	26 (20.6)	209 (13.8)
Intensive care unit	2 (1.6)	229 (15.1)
Isolation ward	24 (19.0)	248 (16.3)
Internal medicine ward	33 (26.2)	497 (32.7)
Surgical ward	10 (7.9)	111 (7.3)
Others	31 (24.6)	224 (14.8)
**Number of beds in your institution**		
0–499 beds	0 (0)	830 (54.7)
500–999 beds	0 (0)	611 (40.3)
1,000–1,499 beds	0 (0)	73 (4.8)
1,500 beds or more	126 (100)	4 (0.3)
Participation in personal protective equipment donning/doffing training before the COVID-19 pandemic	122 (96.8)	1,123 (74.0)
Total working hours per week when psychological distress was the greatest (hours, median, IQR)	45, 40–60	40, 30–46

*IQR, inter-quartile range*.

The average scores for each statement of K10 when psychological distress was the greatest and during the last 30 days of the survey are shown in Table 2. The missing question values were 0%. The total scores (standard deviation) of K10 when psychological distress was the greatest and in the latest 30 days of the survey in Singapore and Japan were 24.45 (8.85) and 20.06 (8.77), respectively. The median period from the peak of psychological distress to the time of this survey in Singapore and Japan was 6 months each (IQR: 5–6 and 4–7, respectively). The potential determinants of psychological distress among frontline nurses and physicians who cared for COVID-19 patients in infectious disease hospitals in Singapore and Japan are shown in Table 3. The missing question values were 0%.

**Table 2:** Average scores for each statement of Kessler Psychological Distress Scale (K10) when psychological distress was the greatest and in the latest 30 days of the survey.

**Questions**	**Average score (standard deviation)**	**Average score (standard deviation)**
	**of the K10 question when**	**of the K10 question in the**
	**psychological distress was the greatest**	**latest 30 days of the survey**
How often did you feel tired out for no good reason?	3.38 (1.09)	2.77 (1.18)
How often did you feel nervous?	3.50 (1.08)	2.54 (1.10)
How often did you feel so nervous that nothing could calm you down?	2.51 (1.20)	1.93 (1.07)
How often did you feel hopeless?	2.26 (1.21)	1.77 (1.07)
How often did you feel restless or fidgety?	2.22 (1.11)	1.80 (0.99)
How often did you feel so restless that you could not sit still?	1.75 (1.03)	1.57 (0.92)
How often did you feel depressed?	2.47 (1.16)	2.03 (1.05)
How often did you feel that everything was an effort?	2.55 (1.16)	2.13 (1.11)
How often did you feel so sad that nothing could cheer you up?	1.92 (1.09)	1.71 (0.98)
How often did you feel worthless?	1.89 (1.12)	1.80 (1.10)
Total score	24.45 (8.85)	20.06 (8.77)

**Table 3:** Potential determinants of the psychological distress of frontline nurses and physicians who cared for COVID-19 patients in hospitals in Singapore and Japan.

**Experiences regarding COVID-19 patient care**	***n* (%)**
I had trouble dealing with panicked patients or patients who did not cooperate with the quarantine.	584 (35.5)
I felt that people were prejudiced against my family and I because I was involved in COVID-19 patient care.	556 (33.8)
My patients died of COVID-19.	440 (26.8)
There was an outbreak of COVID-19 in my hospital.	217 (13.2)
**Clinical practice in daily COVID-19 patient care^a^**	**Average score (standard deviation)**
I practiced hand hygiene.	4.84 (0.40)
I carefully put on and took off my PPE.	4.82 (0.45)
I continued to self-quarantine by being apart from family.	2.32 (1.65)
**Knowledge on COVID-19**	***n*** **(%)**
Health care providers should use an N95 mask or higher-level respirator when performing aerosol generation procedures such as intubation, wiping patients and changing diapers (false).	306 (18.6)
When aerosol-generating procedures are performed and there is a shortage of N95 masks, N95 masks can be reused by covering them with a face shield or a surgical mask (true).	1,119 (68.1)
Once COVID-19 is completely ruled out, airborne and contact prevention and standard precaution can be terminated (false).	1,360 (82.7)
People with COVID-19 are not infectious before symptom onset, however, they often become infectious on the second to third day after the onset of symptoms (false).	1,364 (83.0)
A negative severe acute respiratory syndrome coronavirus 2 (SARS-CoV-2) polymerase chain reaction (PCR) test result can rule out COVID-19 (false).	1,522 (92.6)
SARS-CoV-2 can be detected in respiratory droplets, stools, and sweat in an infected patient (false).	412 (25.1)
Since alcohol is not effective against the new coronavirus, it is important to wash your hands with running water and soap (false).	1,394 (84.8)

a *Average score on a five-point Likert-type scale; 1 point for (i) 0–20%, 2 points for (ii) 21–40%, 3 points for (iii) 41–60%, 4 points for (iv) 61–80%, 5 points for (v) 81–100%*.

### Primary Endpoint

The results of multivariate logistic regression analysis are summarized in Table 4. An increased risk of prolonged psychological distress among frontline nurses and physicians was associated with being a nurse rather than with being a physician (aOR = 2.40, 95% CI: 1.24–4.66), underlying medical condition (aOR = 1.74, 95% CI: 1.22–2.46), experience of prejudice against them and their families because they participated in COVID-19 patient care (aOR = 3.05, 95% CI: 2.23–4.18), experience with panicked patients or patients who did not cooperate with the quarantine (aOR = 2.36, 95% CI: 1.71–3.25), and experience of an outbreak of COVID-19 in the hospital (aOR = 2.05, 95% CI: 1.38–3.04). On the other hand, a decreased risk of prolonged psychological distress among frontline nurses and physicians was associated with larger number of beds in the hospital (aOR = 0.73, 95% CI: 0.57–0.94), clinical practice of carefully putting on and taking off PPE in daily COVID-19 patient care (aOR = 0.52, 95% CI: 0.37–0.73), and knowledge on COVID-19 (aOR = 0.82, 95% CI: 0.72–0.94).

**Table 4:** Multivariate logistic regression analysis of prolonged psychological distress of frontline nurses and physicians.

		**Adjusted odds ratio**	**95% CI**	***p*-value**
Demographic	Age	0.98	0.97–1.00	0.05
characteristics	Being a nurse rather than a physician	2.40	1.24–4.66	0.01
	Underlying medical condition	1.74	1.22–2.46	<0.01
	Number of beds in the hospital^a^	0.73	0.57–0.94	0.01
Experiences regarding COVID-19 patient care	I felt that people were prejudiced against my family and I because I was involved in COVID-19 patient care.	3.05	2.23–4.18	<0.01
	I had trouble dealing with panicked patients or patients who did not cooperate with the quarantine.	2.36	1.71–3.25	<0.01
	There was an outbreak of COVID-19 in the hospital.	2.05	1.38–3.04	<0.01
Clinical practice in daily COVID-19 patient care	I carefully put on and took off my PPE.	0.52	0.37–0.73	<0.01
Knowledge on COVID-19	Knowledge on COVID-19	0.82	0.72–0.94	0.01

*CI, confidence interval*.

a *The number of beds is divided into four categories; 0: 0–499 beds (reference), 1: 500–999 beds, 2: 1,000–1,499 beds, 3: 1,500 beds or more*.

### Secondary Endpoint

**Figure 1:**
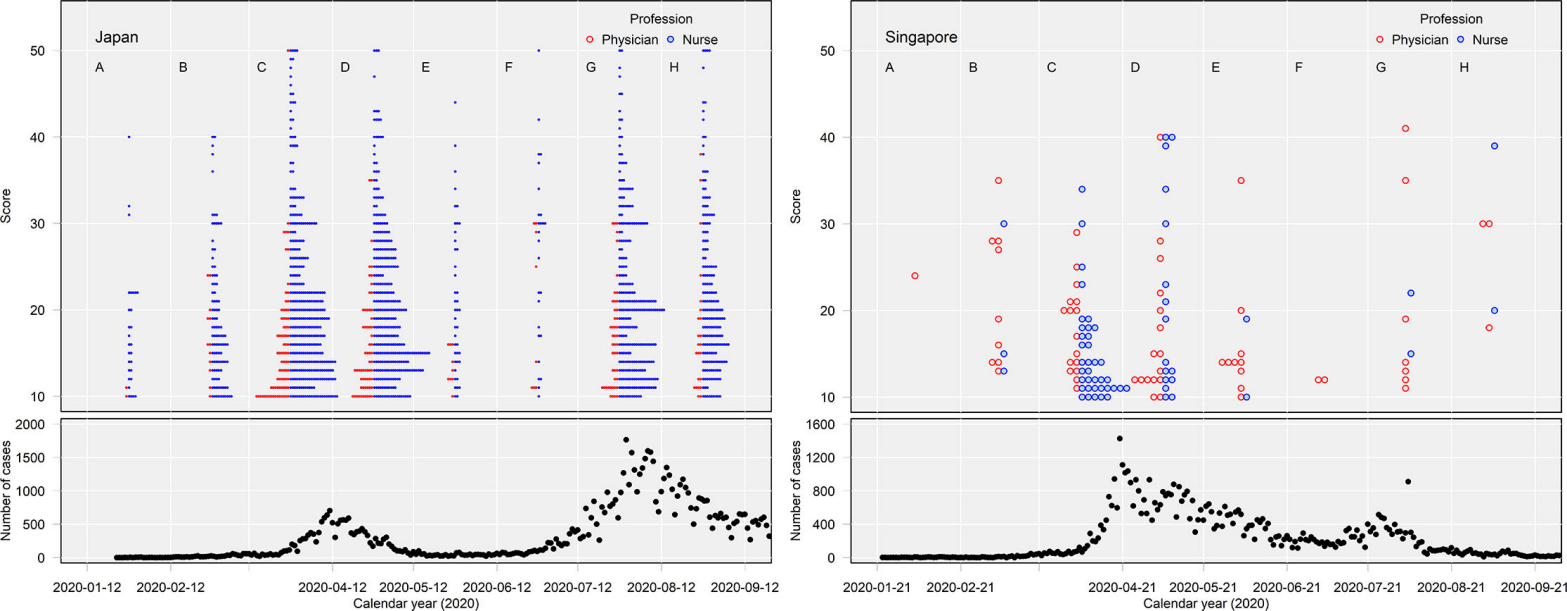
The figure shows that 85 nurses and physicians (67.5%) in Singapore and 788 nurses and physicians (51.9%) in Japan experienced the greatest psychological distress 1 month before and after the 1st peak of the new confirmed cases (periods C and D).

The results of the linear regression analysis are presented in Table 5. Determinants of psychological distress of frontline nurses and physicians included the following: younger age (β = −0.84, *t* = −3.61, *p* = <0.01); being female (β = 0.08, *t* = 2.79, *p* = 0.01); being a nurse rather than a physician (β = 0.16, *t* = 5.28, *p* = < 0.01); presence of underlying medical condition (β = 0.09, *t* = 3.75, *p* = < 0.01); longer working hours (β = 0.10, *t* = 4.41, *p* = < 0.01); not working in internal medicine ward (β = −0.05, *t* = −2.35, *p* = 0.02); working in Japan rather than in Singapore (β = 0.09, *t* = 3.75, *p* = < 0.01); less knowledge on COVID-19 (β = −0.07, *t* = −2.79, *p* = 0.01); experience of prejudice against them and their families because they were involved in COVID-19 patient care (β = 0.22, *t* = 9.77, *p* = < 0.01); experience with panicked patients or patients who did not cooperate with the quarantine (β = 0.19, *t* = 8.39, *p* = < 0.01); and experience of an outbreak of COVID-19 in the hospital (β = 0.11, *t* = 5.11, *p* = < 0.01). The relationship between the number of new confirmed cases and the number of participants whose psychological distress was the greatest was described in Figure 1 which shows that 85 nurses and physicians (67.5%) in Singapore and 788 nurses and physicians (51.9%) in Japan experienced the greatest psychological distress one month before and after the 1st peak of the new confirmed cases.

**Table 5:** Multivariate linear regression analysis of Kessler Psychological Distress Scale score when respondents' psychological distress was the greatest.

		**Unstandardised coefficients**	**Standardized coefficients**		***t***	***p*-value**
		**B**	**Standard error**	**Beta**		
Demographic	Age	−0.07	0.02	−0.08	−3.61	<0.01
characteristics	Being female	1.52	0.55	0.09	2.79	0.01
	Being a nurse rather than a physician	3.54	0.67	0.16	5.28	<0.01
	Underlying medical condition	1.77	0.47	0.09	3.75	<0.01
	Main workplace in internal medicine ward	−1.01	0.43	−0.05	−2.35	0.02
	Working in Japan rather than in Singapore	3.03	0.81	0.09	3.75	<0.01
	Total working hours per week when psychological distress was the greatest	1.65	0.37	0.10	4.41	<0.01
Experiences regarding COVID-19 patient care	I had trouble dealing with panicked patients or patients who did not cooperate with the quarantine.	3.50	0.42	0.19	8.34	<0.01
	I felt that people were prejudiced against my family and I because I was involved in COVID-19 patient care.	4.15	0.43	0.22	9.77	<0.01
	There was an outbreak of COVID-19 in the hospital.	2.99	0.59	0.11	5.11	<0.01
Knowledge on COVID-19	Knowledge on COVID-19	−0.48	0.17	−0.07	−2.79	0.01

## Discussion

This two-country questionnaire survey is one of few studies to identify factors contributing to the prolonged psychological distress of frontline nurses and physicians who took care of COVID-19 patients in hospitals in Singapore and Japan.

The first important finding was that the increased risk of prolonged psychological distress of frontline nurses and physicians was associated with being a nurse, underlying medical condition, smaller number of beds in the hospital, and a COVID-19 outbreak in the hospital. In addition, a decreased risk of prolonged psychological distress was associated with age, indicating that younger nurses and physicians tended to have prolonged psychological distress. This could help to identify and prioritize healthcare providers who require mental care during the COVID-19 pandemic. Furthermore, this identification of factors is important for mitigating and preventing prolonged psychological distress.

In non-healthcare settings, younger people reported a significantly higher prevalence of generalized anxiety disorder and depressive symptoms (21) and reported more vulnerability regarding their mental health conditions (22). Young people can be stressed easily as they collect information from social media (22) or are vulnerable to loneliness or lack of family support (10, 23). A healthcare study found that single physicians had a higher risk of developing psychiatric symptoms than married nurses in the acute respiratory syndrome (SARS) epidemic (24). The current study hints at a similar finding, with young nurses and physicians tending to experience prolonged psychological distress. Loneliness or a sense of isolation among young single healthcare providers may contribute to this; however, further studies are required to explore and clarify its mechanism.

Second, the increased risk of prolonged psychological distress among participants could be related to people being prejudiced against them and their families because they were engaged in COVID-19 patient care. Healthcare facilities were considered epicenters, which triggered widespread irrational prejudice and discrimination against healthcare providers (25). They were denied use of public buses and taxis and were even urged to vacate rental housing. Family members also suffered defamation. During the influenza H1N1 pandemic in 2009, discrimination against frontline health care workers was acknowledged as a public health crisis (26). To continue the fight against COVID-19, public leaders must take responsibility for front-line healthcare providers' safety, show strong solidarity toward them, and prepare effective communication strategies to tackle discrimination and social sanctions against them (25).

Third, several factors reduced the risk of prolonged psychological distress among frontline nurses and physicians; this was associated with the clinical practice of carefully wearing and removing PPE in daily COVID-19 patient care and having adequate knowledge on COVID-19. In previous reports, protective factors against greater psychological distress included having sufficient local medical resources (27); taking precautionary measures (e.g., hand hygiene, wearing masks); and having current, accurate health information (e.g., treatment, local outbreak situation) (11). This implies that adequate medical resources and education on precautionary measures, including basic standard measures, are critical for mitigating prolonged psychological distress. They are also necessary measures for outbreak prevention, especially in smaller hospitals with scarce medical resources and infectious diseases specialists.

Fourth, determinants of peaked psychological distress among frontline nurses and physicians included being female, longer working hours, not working in the internal medicine ward, and working in Japan rather than in Singapore. Additionally, 67.5% of nurses and physicians in Singapore and 51.9% in Japan had their psychological distress peak 1 month before and after the first peak of the new confirmed cases. This result was consistent with previous reports demonstrating that being female (11, 28), being nurses (11, 28) and longer working hours (29) were associated with a high risk of mental health problems. Being a nurse and being female also appeared to confer a high risk of acquiring trauma or stress-related disorders, depression, and anxiety in other viral epidemics such as SARS, Ebola virus disease, and Middle East Respiratory Syndrome (28). This may help us identify a vulnerable target population that could benefit from the outreach efforts of psychologists/psychiatrists/social workers for defining the appropriate timing of mental care in future epidemics or pandemics. This study showed that frontline nurses and physicians in Japan experienced more prolonged psychological distress compared to those in Singapore. This may be because the mortality rate between January 2020 and September 2020 in Japan (1.88%) was higher than that in Singapore (0.05%), indicating that the frontline nurses and physicians in Japan needed to take care of dying patients and interact with the families of deceased patients more often (30). Management may also have affected the levels of psychological distress, as a previous study revealed that higher levels of psychological distress were associated with COVID-19 procedural management (31). Finally, differences in culture, medical systems, and demographic factors of participating healthcare providers in each country may have contributed to this disparity.

Despite its strengths, this study has several limitations. First, this study was prone to recall bias. Second, the response rate only reached 23.8%, although we minimized the number of questions and the respondents' distress and sent two reminders to potential participants. Third, we did not formally test the validity and reliability of the questionnaire items. Furthermore, this study could not investigate socioeconomic status (11), social support (32), and working in the hardest-hit area (1) as potential determinants that contributed to the psychological distress of frontline nurses and physicians. However, established tools to measure such variables are not yet available, and we performed an exploratory analysis. Fourth, this study is prone to response bias because of the self-report survey. Fifth, we could identify the risk factors for 1 year; however, further studies are needed to identify them for longer periods in this lengthy pandemic. Sixth, there was an imbalance between the number of participants in Singapore and Japan. In addition to this, an online self-report questionnaire was sent to eligible nurses and physicians in 370 hospitals in Japan and only one hospital in Singapore. This may have influenced the results. Finally, the participants in this study were limited to frontline nurses and physicians who cared for patients with COVID-19 in hospitals in Singapore and Japan. Multiple factors (e.g., cultural differences) may have contributed to psychological distress in each country. Additionally, the findings of this study may not be generalisable to other ethnic groups because of the multiple influencing factors.

In conclusion, this survey identified factors that contributed to the prolonged psychological distress of frontline nurses and physicians involved in the care of patients with COVID-19 in hospitals in Singapore and Japan. This identification of factors could help us seek out vulnerable healthcare providers who need urgent mental care during the COVID-19 pandemic. Multiple measures, including adequate medical resources, education on precautionary measures, and effective communication strategies tackling discrimination and social sanctions against front-line healthcare providers are critical to mitigate and prevent prolonged psychological distress.

## Data Availability Statement

The original contributions presented in the study are included in the article/Supplementary Material, further inquiries can be directed to the corresponding author/s.

## Ethics Statement

The Singhealth Centralized Institutional Review Board approved the study (2020/2807) in Singapore, and the Institutional Review Board at the National Center for Global Health and Medicine approved the study (NCGM-G-003562-00) in Japan. The patients/participants provided their written informed consent to participate in this study.

## Author Contributions

SM: conceptualization, methodology, formal analysis, investigation, data curation, writing—original draft preparation, writing review and editing, visualization, supervision, and project administration. BT: conceptualization, methodology, data curation, and writing review and editing. HK: conceptualization, methodology, formal analysis, and investigation. YA: methodology, formal analysis, and investigation. TS: data curation and writing review and editing. SA: data curation. SK and KH: conceptualization, methodology, and writing review and editing. SS and TT: conceptualization and writing review and editing. EK: writing review and editing and supervision. NO: conceptualization, methodology, formal analysis, investigation, writing review and editing, and supervision. All authors contributed to the article and approved the submitted version.

## Conflict of Interest

The authors declare that the research was conducted in the absence of any commercial or financial relationships that could be construed as a potential conflict of interest.

## Publisher's Note

All claims expressed in this article are solely those of the authors and do not necessarily represent those of their affiliated organizations, or those of the publisher, the editors and the reviewers. Any product that may be evaluated in this article, or claim that may be made by its manufacturer, is not guaranteed or endorsed by the publisher.
