# Supplementary material for: Factors Associated With Prolonged Psychological Distress Among Nurses and Physicians Engaged in COVID-19 Patient Care in Singapore and Japan
Source: Front Psychiatry. 2022 Apr 28;13:781796. doi: 10.3389/fpsyt.2022.781796 (PMC9096717; doi:10.3389/fpsyt.2022.781796)
Supplement: Supplementary file 1 [file Data_Sheet_1.PDF]

The principal component analysis to identify the underlying structure of knowledge on COVID-19

**Variables**	**Component 1**	**Component 2**	**Component 3**
Health care providers should use an N95 mask or higher-level respirator when performing aerosol generation procedures such as intubation, wiping patients and changing diapers (false).	.507	.512	.101
When aerosol-generating procedures are performed and there is a shortage of N95 masks, N95 masks can be reused by covering them with a face shield or a surgical mask (true).	.149	-.243	.807
Once COVID-19 is completely ruled out, airborne and contact prevention and standard precaution can be terminated (false).	.479	-.279	-.500
People with COVID-19 are not infectious before symptom onset, however, they often become infectious on the second to third day after the onset of symptoms (false).	.598	-.182	-.011
A negative severe acute respiratory syndrome coronavirus 2 (SARS-CoV-2) polymerase chain reaction (PCR) test result can rule out COVID-19 (false).	.569	-.356	-.136
SARS-CoV-2 can be detected in respiratory droplets, stools, and sweat in an infected patient (false).	.266	.756	-.077
Since alcohol is not effective against the new coronavirus, it is important to wash your hands with running water and soap (false).	.536	.039	.321
